# Fecal Supernatant from Adult with Autism Spectrum Disorder Alters Digestive Functions, Intestinal Epithelial Barrier, and Enteric Nervous System

**DOI:** 10.3390/microorganisms9081723

**Published:** 2021-08-13

**Authors:** Jacques Gonzales, Justine Marchix, Laetitia Aymeric, Catherine Le Berre-Scoul, Johanna Zoppi, Philippe Bordron, Marie Burel, Laetitia Davidovic, Jean-Romain Richard, Alexandru Gaman, Florian Lejuste, Julie Z. Brouillet, Françoise Le Vacon, Samuel Chaffron, Marion Leboyer, Hélène Boudin, Michel Neunlist

**Affiliations:** 1 Inserm, TENS, The Enteric Nervous System in Gut and Brain Disorders, IMAD, Université deNantes, 44035 Nantes, France; jacques.gonzales@etu.univ-nantes.fr (J.G.); justine.marchix@univ-nantes.fr (J.M.); laetitia.aymeric@univ-angers.fr (L.A.); catherine.scoul@univ-nantes.fr (C.L.B.-S.); johanna.zoppi@etu.univ-nantes.fr (J.Z.); philippe.bordron@univ-nantes.fr (P.B.); gloussou@yahoo.com (M.B.); 2 Department of Biology, Université d’Angers, 44045 Angers, France; 3 Centre National de la Recherche Scientifique, Institut de Pharmacologie Moléculaire et Cellulaire, 06560 Valbonne, France; davidovic@ipmc.cnrs.fr; 4 Department of Life Science, Université Côte d’Azur, 06103 Nice, France; 5 Fondation FondaMental, 94000 Créteil, France; gaman.alexandru@gmail.com (A.G.); flejuste@gmail.com (F.L.); marion.leboyer@inserm.fr (M.L.); 6 INSERM U955 Neuro-Psychiatrie Translationnelle, Institut Mondor de Recherche Biomédicale, 94010 Créteil, France; Jean-Romain.Richard@inserm.fr (J.-R.R.); julie.brouillet.z@gmail.com (J.Z.B.); 7 AP-HP, Département médico-Universitaire d’Addictologie et de Psychiatrie des Hôpitaux Henri Mondor, Université Paris Est Créteil, 94010 Créteil, France; 8 BioFortis Mérieux NutriSciences, 44800 Saint-Herblain, France; francoise.le.vacon@mxns.com; 9 CNRS UMR 6004, Laboratoire des Sciences du Numérique de Nantes (LS2N), Université de Nantes, 44000 Nantes, France; samuel.chaffron@univ-nantes.fr

**Keywords:** microbiota, enteric nervous system, intestinal permeability, bacterial metabolite, autism

## Abstract

Autism Spectrum Disorders (ASDs) are neurodevelopmental disorders defined by impaired social interactions and communication with repetitive behaviors, activities, or interests. Gastrointestinal (GI) disturbances and gut microbiota dysbiosis are frequently associated with ASD in childhood. However, it is not known whether microbiota dysbiosis in ASD patients also occurs in adulthood. Further, the consequences of altered gut microbiota on digestive functions and the enteric nervous system (ENS) remain unexplored. Therefore, we studied, in mice, the ability offecal supernatant (FS) from adult ASD patients to induce GI dysfunctions and ENS remodeling. First, the analyses of the fecal microbiota composition in adult ASD patients indicated a reduced α-diversity and increased abundance of three bacterial 16S rRNA gene amplicon sequence variants compared to healthy controls (HC). The transfer of FS from ASD patients (FS–ASD) to mice decreased colonic barrier permeability by 29% and 58% compared to FS–HC for paracellular and transcellular permeability, respectively. These effects are associated with the reduced expression of the tight junction proteins JAM-A, ZO-2, cingulin, and proinflammatory cytokines TNFα and IL1β. In addition, the expression of glial and neuronal molecules was reduced by FS–ASD as compared to FS-HC in particular for those involved in neuronal connectivity (βIII-tubulin and synapsin decreased by 31% and 67%, respectively). Our data suggest that changes in microbiota composition in ASD may contribute to GI alterations, and in part, via ENS remodeling.

## 1. Introduction

Autism spectrum disorder (ASD) is a heterogeneous group of neuro-developmental disorders causing life-long impairments defined by a lack of social skills, empathy, communication deficits, andpatterns of repetitive behaviors and restricted interests. The prevalence of ASD has dramatically increased in the world, rising from 0.02% in 1975 to approximately 1% of the general child population [1,2,3]. Although ASD is primarily considered a brain disorder [4,5,6,7], peripheral systems and organs are also affected, and, in particular, the gastrointestinal (GI) tract [8]. Indeed, GI symptoms in children with ASD are 4.4 times more prevalent than for neurotypical children, with higher rates of constipation, diarrhea, and abdominal pain [9]. Several studies indicated increased intestinal permeability in approximately 40% of ASD patients, although others showed no modification [10,11,12,13]. In another study, duodenal biopsies from ASD children showed the altered expression of tight junction proteins which are key components for intestinal epithelial barrier integrity [14]. Moreover, a positive correlation between the severity of autistic and GI symptoms was reported [15,16,17], further supporting the existence of the coincident brain and gut dysfunctions in ASD. GI dysfunctions associated with ASD conditions are also observed in ASD animal models in which defects in colonic transit and permeability were reported along with altered behavior [18,19,20]. Despite the central importance of the enteric nervous system (ENS) in the control of GI functions, little is known about ENS structural or functional modifications in ASD, which could act as an underlying mechanism for GI dysfunctions in ASD. The ENS is a complex network of enteric neurons and glia, organized as interconnected ganglia distributed all along the digestive tract. [21,22]. Abnormalities in ENS structure, such as changes in the number of enteric neurons and in the proportion of neuronal sub-types, were described in genetic ASD animal models [18,19,23,24]. However, these genetic models failed to entirely reflect the underlying complexity of the interactions between genetic and environmental factors in ASD. In particular, the role of the gut microbiota as a contributor to ENS remodeling in ASD currently remains unknown.

Gut microbiota is increasingly recognized as a central contributor to gut and brain health and diseases [25]. In particular, in neurodegenerative diseases, recent studies reported altered microbiota composition in Parkinson’s disease (PD) patients that could contribute to intestinal inflammatory conditions observed in PD patients [26]. Furthermore, the transfer of feces of PD patients (as compared to healthy donors) enhanced motor symptoms in genetically PD-prone mice [27] further highlighting the putative ‘pathogenic’ potential of gut microbiota in neurodegenerative diseases. Among putative mechanisms linking microbiota to PD progression, is the fact that the altered microbiota composition, in particular increased gram-negative bacteria found in PD, could contribute to the induction of an LPS-dependent inflammatory response of the gut and the brain leading to enhanced motor symptoms and dopaminergic damage [28,29]. Further supporting the role of inflammation in neurodegenerative development is the fact that chronic oxidative stress plays a central role in the progression of PD [30]. Concerning ASD, several studies reported changes in the composition of fecal microbiota and bacteria-derived metabolites in ASD patients compared to neurotypical individuals [17,31,32,33,34,35,36]. However, these studies focused on pediatric or adolescent populations and no data are so far available for adult ASD patients [20,37,38,39]. Furthermore, a causal role of microbiota in ASD symptoms has recently been suggested by showing that the transplantation of fecal microbiota from ASD children to mice can induce core behavioral ASD symptoms [40,41]. However, whether gut microbiota of ASD patients can impact GI and ENS functions and modulate gut inflammatory and oxidative stress response remain to be determined. Recent studies have highlighted the ability of gut microbiota to contribute to ENS functions, especially during the perinatal period [42]. For instance, gut microbiota regulates the number and phenotype of enteric neurons and glial cells [43,44,45], as well as the neuronal transcriptional profile [46]. This is possibly mediated via bacterial metabolites, including short chain fatty acids (SCFA), such as butyrate [47,48] or bacterial membrane components [49]. However, the ability of microbiota from ASD patients to contribute directly to ENS remodeling currently remains unknown.

Altogether, the aims of the present study were two-fold. First, we aimed to determine whether microbiota composition was altered in adult ASD patients in comparison to age-matched healthy controls. Second, we aimed to determine whether fecal supernatants from ASD patients, as compared to healthy controls, could induce changes in GI functions and ENS remodeling.

## 2. Materials and Methods

### 2.1. Ethics Statement and Study Approval

Participants with ASD were recruited through the Autism Expert Center (FondaMental foundation), Chenevier–Mondor Hospital in Créteil, France. This study received approval on 02 February 2016 from the French Ethical Committee for Biomedical Research (Comité de Protection des Personnes Ile de France V, protocole 2013-A01286-39 registered under no. 16113 with the collection code DC 2009-930. Human samples were collected after obtaining written informed consent from the patients or the legal guardians prior to inclusion in the study. Participants were screened for eligibility criteria through questionnaires, if the criteria were met, sample collection kits were given to them. The healthy controls (HC) samples come from the BIOSAINE and GOMMS projects as a part of the biobank of Biofortis (Mérieux NutriSciences, Saint-Herblain, France) The biocollections are registered at the French Research Ministry (CEBH): under NO. CODECOH:AC-2013-1792 and AC-2014-2232.

C57BL/6RJ SPF mice were obtained from the Janvier Laboratory (Le Genest Saint-Isle, France) and were housed in a 12 h light-dark cycle with free access to water and food. The experimental protocols were performed in accordance with the recommendations of the Animal Care and Use Committee of Nantes (France) and were approved by the French Ministry of Research (Agreement NO. 21485-201907151349898 v2, approved 10 April 2019).

### 2.2. Subjects, Feces Collection, and Preparation of Fecal Supernatant (FS)

**Figure 1:**
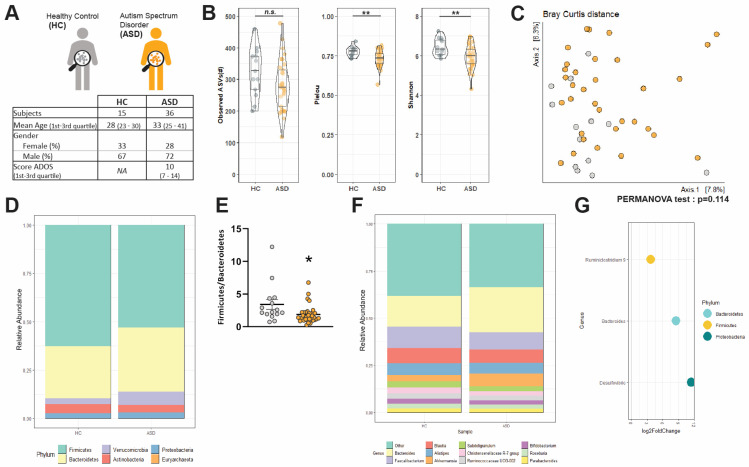
Fecal microbiota composition of HC and ASD patients. (**A**) Summary of demographic characteristics of HC and ASD patients. (**B**) Alpha diversity in HC and ASD individuals was estimated by the richness (Observed ASVs), evenness (Pielou index), and Shannon index, n.s.: non-significant, ** *p* < 0.01, (**C**) Principal coordinate analysis (PCoA) of bacterial beta-diversity in HC and ASD individuals generated by subsampling and Bray–Curtis distance. (**D**) Bacterial taxonomic profile in HC and ASD patients at phylum level. (**E**) *Firmicutes*: *Bacteroidetes* ratio in HC and ASD individuals. Values are represented as mean ± SEM (HC: *n* = 13–15, ASD: *n* = 34–36). Statistical analyses were performed with the Mann–Whitney test, * *p* < 0.05. (**F**) Bacterial taxonomic profile in HC and ASD patients at genus level (top 11 most abundant genus). (**G**) Genera significantly increased in ASD compared to HC individuals according to DESeq2 analysis. Data are presented as Log2 of the fold-of-change (FC) between ASD and HC.

A group of fifteen HC (ten men, five women) was included with the inclusion criteria based on stool frequency (less than three per day but more than three per week). The exclusion criteria were eating disorders and gastrointestinal disorders (constipation, excessive flatulence, irritable bowel syndrome, Crohn’s disease, ulcerative colitis). Thirty-eight ASD adults (twenty-six men, twelve women) received a clinical diagnosis of Asperger syndrome or autism without mental retardation according to the Diagnostic and Statistical Manual of Mental-4-revised (DSM-IV R) criteria and the Autism Diagnostic Observation Schedule (ADOS) scores (Figure 1A).The diagnosis was confirmed by interviews with parents or caregivers using the Autism Diagnostic Interview-Revised (ADI-R). Subjects were not eligible for the study if they had been on antibiotics or antifungal agents within the preceding eight weeks. HC and ASD individuals were aged between 18 and 50 years old at the time of feces collection (Figure 1A). Feces from ASD individuals were collected by individuals at home with the GENboxanaer kit (ref 96124, Biomérieux, Marcy l’Etoile, France), stored immediately at 4 °C, and brought to the Centre d’Investigation Clinique of Créteil hospital in less than 24 h. Feces from HC were collected by the individuals at home in a collect-kit, immediately stored at 4 °C, and brought to the Biofortis company in less than 48 h. Each sample was aliquoted by a 1g tube and stored at −80 °C before use. For FS preparation, feces were thawed on ice, homogenized in Hanks Balanced Salt Solution (HBSS; 125 mg/mL), and centrifuged at 3800× *g* for 15 min at 4 °C. Supernatants were filtered through a 20 µm cell strainer and then centrifuged at 13,000× *g* for 15 min at 4 °C. The resulting fecal supernatant (FS) was then filtered through a 0.22 µm filter and stored at −80 °C.

### 2.3. Microbiota and Bacterial Metabolite Analysis

#### 2.3.1. 16S rRNA Gene Sequencing

Microbial genomic DNA was extracted from frozen mouse feces using the Maxwell^®^ 16 instrument. The sequencing library was generated using the Nextera XT Index kit (Illumina, Paris, France). The V3–V4 regions of the 16S rRNA gene were amplified by PCR using the universal primers 341F/805R. Paired-end (2 × 250 PE) sequencing of PCR products was performed using the Illumina MiSeq platform (MiSeq V2 reagent kit; Illumina Inc., San Diego, CA, USA).

#### 2.3.2. Data Processing

Raw reads were processed using microSysMics (https://bio.tools/microSysMics, 20 April 2020), a workflow that relies on the Quantitative Insights into Microbial Ecology 2 (Qiime2) toolbox [50]. Calculations were performed on the BiRD platform (Nantes). The R package DADA2 v1.14 was used to demultiplex, quality filter, chimera filter, denoise the sequence reads, and call amplicon sequence variants (ASVs) [51]. A phylogenetic tree was generated against the SILVA v132 reference database using FastTree and MAFFTalignment in Qiime2. Diversity metrics were calculated on the rarefied ASV matrix. We chose a subsampling depth of 34,610 sequences per sample, for a final rarefied dataset of 47 samples (>90% of samples, 13 HC-34 ASD). Alpha diversity was visualized and estimated by the metrics of observed ASVs, Pielou’s and Shannon’s indexes, using R packages dplyr v0.8.5, ggplot2 v3.3.0 and ggstatsplot v0.4.0. Beta-diversity ordinations were calculated on the Bray–Curtis dissimilarity matrix, Jaccard distance, and (un)weighted UniFrac distances, and PCoA were visualized using phyloseq v1.30.0 and ggplot2 packages. The difference in microbial β-diversity between classes was tested using permutational multivariate analysis of variance (PERMANOVA) using vegan 2.5–6. The relative abundance of microbial ASVs was calculated from the nonrarefied ASV matrix. ASVs were filtered based on a prevalence of 20%. Community structure and metadata factors that influence it were studied by using omeClust 1.1.8 [52] on the Bray–Curtis dissimilarity matrix.Differential abundance analysis was performed using Phyloseq v1.30.0 and DESeq2 v1.26 [53]. The estimation of size factor was set to use “poscounts” as suggested for microbiota analysis. Only results displaying an adjusted *p*-value below 0.05 after Benjamini–Hochberg correction (false discovery rate, FDR) are reported and visualized using ggplot2. Difference in the relative CLR-transformed abundance of the top 11 genera between HC and ASD was tested using the non-parametric Wilcoxon sign rank test.

#### 2.3.3. Bacterial Metabolite Analysis

SCFA were analyzed in FS by gas chromatography–mass spectrometry (GC–MS) as previously described [54]. Bile acids (BA) were analyzed by ultra-performance liquid chromatography–tandem mass spectrophotometer (UPLC–MS/MS) as previously described [55].

### 2.4. Microbiota-Metabolite Association

Heatmaps were generated to assess the correlation between microbiota-derived metabolites and genuslevel taxa in HC and ASD. ASVs were aggregated to the genus-level. Samples with missing metabolite concentration values were removed and a dataset of 14 HC and 28 ASD was used for the correlation analysis. Only the genera and metabolites seen in at least 30% of the samples per group (HC and ASD) were considered. A heatmap matrix was generated from spearman correlations and hierarchical clustering was used to cluster genera separately for HC and ASD using tidyverse v1.3.0, phyloseq v1.30.0, and microbiome v1.8.0 packages. Heatmaps and statistical analysis were generated using the associate function of the microbiome package. Only results displaying an adjusted p-value below 0.05 after Benjamini–Hochberg correction are reported.

### 2.5. Mice Model of Fecal Supernatant Administration

Male C57BL/6RJ SPF mice of 7 weeks of age (Janvier Laboratory, France) received, for 15 days, a mix of antibiotics and antifungals containing 100 µg/g metronidazole, vancomycin (50 µg/g), neomycin (100 µg/g), and amphotericin B (1 µg/g) by oral gavage and 1 g/L ampicillin through the drinking water. At the end of antibiotic treatment, enemas of 20 µL/g of animal weight were performed with FS from HC (FS–HC, one mouse per stool donor, *n* = 10 donors) or from ASD individuals (FS–ASD, one mouse per stool donor, *n* = 12 donors) using a 24-gauge cannula introduced in the rectum up to 5 cm. Mice received an enema every 12 h for 48 h (a total of 5 enemas per mouse).

### 2.6. In Vivo Intestinal Motility Assay

For the fecal pellet output (FPO), mice were placed alone in a cage without bedding, food, or water. The fecal pellets were harvested for 2 h. Fecal water content corresponded to the weight difference between wet and dry pellets. To assess the rate of transit time, mice received by gavage a solution containing 60 mg/mL of carmine red (5 µL/g of animal weight) and were euthanized 2 h later. The total length of the intestine and the distance between the jejunum entry and the carmine red migration front were measured.

### 2.7. Intestinal Permeability Assay

#### 2.7.1. In Vivo Intestinal Permeability

Mice received, by oral gavage, 5 µL/g of the animal weight of a solution containing 10 mg/mL of fluorescein-5.6 sulfonic acid (FSA, Thermo Fisher Scientific, Illkirch-Graffenstaden, France) and horseradish peroxidase (HRP, Sigma-Aldrich, Saint-Quentin-Fallavier, France) diluted in 0.5% of carboxy-methyl-cellulose. Blood was collected from the tail vein 2 h before and afterthe gavage. Plasma was isolated by centrifugation at 3200 rpm for 10 min andFSA concentration was determined by measuring the fluorescence intensity at 488 nm using a spectrofluorometer (Varioskan, Thermo Fisher Scientific, Illkirch-Graffenstaden, France). HRP activity was determined by enzymatic assay with tetramethylbenzidine (TMB) substrate (BD Biosciences).

#### 2.7.2. Ex Vivo Intestinal Permeability

Mice were anesthetized with isoflurane prior to cervical dislocation. The colon was removed, and segments of proximal and distal colon were mounted in Ussing chambers (Easy Mount, Warner Instrument, Hamden, CT, USA) in 2 mL of Dulbecco’s Modified Eagle Medium/Nutrient Mixture F-12 (Thermo Fisher Scientific, Illkirch-Graffenstaden, France) maintained at 37 °C and bubbled with a gas flow of 95% O_2_/5% CO_2_. After the addition of FSA and HRP (final concentration 0.1 and 0.375 mg/mL, respectively), permeability was assessed by aliquots taken from the basolateral side at 30 min intervals over a period of 150 min, and the concentration of FSA and HRP were measured, respectively, with a spectrofluorometer and by enzymatic assay with TMB substrate.

### 2.8. ENS Primary Cultures and Treatment

The primary culture of rat ENS was generated from embryonic day 15 (E15) rat intestine as previously described [56]. Briefly, after cell dissociation, the cells were plated at a density of 2.4 × 10^5^ cells/cm^2^ in DMEM/F12 containing penicillin/streptavidin and 10% fetal bovine serum. After 24 h, the medium was replaced by DMEM/F12 containing 1% of N-2 supplement (Invitrogen), and primary cultures were maintained for 11 days. On day 9, cultures were treated for 48 h with FS–ASD or FS–HC (1:500) and were collected in RA1 lysis buffer (Macherey-Nagel) and stored at −80 °C. The toxicity of FS on ENS culture was evaluated by a mitochondrial viability test (MTT assay), which indicated 100% cell survival at the end of FS treatment in comparison to cultures treated with HBSS.

### 2.9. mRNA and Proteins Extraction

Murine colonic tissues, or ENS cultures, were lysed in RA1 buffer with the “Precellys 24” tissue homogenizer (Bertin Technologies, Montigny-le-Bretonneux, France). The lysate was transferred to a Nucleospin RNA II filters column (Macherey Nagel, Hoerdt, France). The proteins were recovered from the flow-through and stored at −20 °C. The RNAs were eluted from the columns and treated with DNase according to the manufacturer’s recommendations and stored at −20 °C.

### 2.10. Quantitative Real-Time PCR (RT-qPCR)

RNA was converted to cDNA using the SuperScript III Reverse Transcriptase (Life Technologies). qPCR was performed on 8 ng RNA equivalent using a StepOnePlus Real-Time PCR Instrument (Life Technologies) with a FastSYBR Green Master Mix kit (Applied Biosystems, Foster City, CA, USA) using the primer pairs listed in Additional File 1 Table S1. Ribosomal protein S6 (RPS6) transcript was used as a reference. The relative expression of the gene of interest was measured by the 2^−ΔΔCt^ method.

### 2.11. Western Blot

Proteins were resuspended in 1:1 protein solving buffer and tris(2-carboxyethyl) phosphine-reducing agent (PSB-TCEP, Macherey-Nagel), sonicated, and denatured for 5 min at 95 °C. Either 5 µg (ENS culture) or 20 µg (colon) of proteins were separated using NuPAGE^TM^ 4–12% Bis-Tris gels (Life Technologies) and transferred to nitrocellulose membranes (Life Technologies). Membranes were incubated overnight at 4 °C with the primary antibodies (listed in Additional File 2 Table S2) Then, with HRP-conjugated anti-rabbit (Life technologies, 1:5000) or anti-mouse secondary antibodies (Sigma, 1:5000) and Clarity Western ECL Substrate (Bio-Rad, Marnes-La-Coquette, France) chemiluminescence of blots was imaged using a laser-scanning densitometry ChemiDoc MP Imaging System (Bio-Rad). Western blot data are expressed as relative values to β-actin normalized to the control mean. Contrasts of the illustrative Western blot have been adjusted with ImageLab software v6 (Bio-Rad).

### 2.12. Histological Analysis

Murine colonic tissues were fixed with 4% paraformaldehyde in 0.1 M phosphate-buffered saline for 3 h at RT, embedded in paraffin, microtome-sectioned (3 μm thick), mounted on glass slides and stained with hemalun, phloxin, and saffron (HPS). Images were acquired with a slide scanner (Hamamatsu Nanozoomer 2.0HT, Massy, France) and viewer software (NDPViewer). Colonic tissue integrity was monitored by scoring the mucosal architecture, cellular infiltration, and muscle thickening, as 0, 1, 2, 3 (from best to less preserved). Multiplication of the score by 1, 2, 3, or 4 was applied if the observed damage covered 25%, 50%, 75%, or 100%, respectively, of the analyzed tissue fragment.

### 2.13. Statistical Analysis

Values are expressed as means ± standard error of the mean (SEM). Outliers were identified using the ROUT method of GraphPad Prism 7.0 with Q = 1%. Group comparison was made using either the Mann–Whitney U-test or the Student t-test when data came from a Gaussian distribution and had no significant difference in their variances. The Gaussian distribution (normality) was evaluated by the Agostino–Pearson omnibus normality test, and the comparison of variances with the Fisher F-test. Differences were considered statistically significant when *p* < 0.05.

## 3. Results

### 3.1. Socio-Demographic Data

Thirty-six adults with ASD without mental retardation were included (ten women, twenty-six men). They were aged 33 ± 1 year on average and their score at the ADOS was 10 ± 0.7 (Figure 1A). Fifteen healthy controls (HC) were included (five women, ten men). They were aged 28 ± 2 years on average (Figure 1A). The two groups were statistically similar with respect to age and sex distribution according to the Student *t*-test (*p* = 0.61) or Fisher exact test (*p* = 0.74).

### 3.2. Changes in α-Diversityand Bacterial Abundance in the Fecal Microbiota of Adult ASD Patients

Ordinations of diversity metrics were used to compare the composition of the fecal microbiota of HC and ASD (Figure 1B,C, Additional File 3 Figure S1). Alpha diversity of HC and ASD microbiota was estimated according to its richness (observed ASVs), evenness (Pielou index), or both (Shannon index) (Figure 1B). Microbiota richness was similar in both HC and ASD individuals (Figure 1B, *p* = 0.09). In contrast, ASD patients showed a reduced evenness (Figure 1B, *p* < 0.01) and a reduced Shannon index (Figure 1B, *p* < 0.01). However, β-diversity was similar between the two groups (Figure 1C, Additional File 3 Figure S1A–C). OmeClust produced six different clusters (Additional File 3 Figure S1D), but none is organized according to the group (i.e, HC vs. ASD) or the sex as indicated by the low Normalized Mutual Information (NMI) scores that characterized the influence of metadata (Additional File 3 Figure S1E).

Next, we identified the taxonomic composition of the gut microbiota in HC and ASD patients. The bacterial communities of HC and ASD were dominated by two main phyla, *Bacteroidetes* and *Firmicutes,* while the phyla *Actinobacteria*, *Proteobacteria,* and *Verrucomicrobia* were less represented (Figure 1D, Additional File 3 Figure S1F). HC and ASD patients exhibited similar abundances of the most represented phyla (Figure 1D, Additional File 3 Figure S1F) overall. However, a significant decrease in the *Firmicutes:Bacteroidetes* ratio was observed in ASD patients as compared to HC (Figure 1E, *p* = 0.01). At the genus level, HC and ASD patients exhibited similar major genera with *Bacteroides*, *Faecalibacterium, Blautia*, *Alistipes*, and *Akkermansia* as the most abundant genera (Figure 1F, Additional File 3 Figure S1G). However, *Bacteroides* were more abundant in ASD patients than in HC but with only borderline statistical significance (*p* = 0.05; Figure 1F, Additional File 3 Figure S1G–H). A differential abundance analysis of ASVs between HC and ASD using DESeq2 identified three ASVs differentially represented between HC and ASD. Those ASVs belonging to *Ruminiclostridium, Bacteroides,* and *Desulfovibrio* genera (from the *Firmicutes*, *Bacteroidetes,* and *Proteobacteria* phyla, respectively) were more abundant in ASD individuals compared to HC (Figure 1G). No downregulated ASVs were observed in ASD compared to HC.

### 3.3. Short Chain Fatty Acids and Bile Acids Are Unchanged in FS–ASD as Compared to FS–HC

Altered microbiota composition in children with ASD can be accompanied by changes in fecal levels of microbiota-derived metabolites, especially the SCFA [15,33,57]. We, therefore, quantified the SCFA in FS from HC and ASD individuals. We also analyzed several BA, previously identified as dysregulated in the BTBR mouse model of idiopathic ASD [18], but, yet never investigated in humans. The concentration of the SCFA acetate, butyrate, propionate, isobutyrate and valerate was not different between the two groups (Table 1). No change was observed for the primary BA, cholic acid (CA), and chenodeoxycholic acid (CDCA), nor the secondary BA, lithocholic acid (LCA), and ursodeoxycholic acid (UDCA). However, there was a trend towards an elevated concentration of deoxycholic acid (DCA) in FS–ASD compared to FS–HC samples (*p* = 0.057).

Next, correlation analysis between the relative abundance of bacterial genera and metabolite concentrations showed a distinct correlation profile in HC and ASD patients (Additional File 4 Figure S2). While no significant correlation was found in HC, several associations between SCFA or BA, and specific bacterial genera were observed in ASD patients. Regarding SCFA, acetate and butyrate concentrations were positively correlated with *Roseburia,* while isobutyrate showed a negative correlation with *(Ruminococcus)Gauvreauiigroup*. Regarding the primary BA, CA was correlated positively with *Dorea*, *Blautia* and negatively with *ChristensenellaceaeR-7 group*, *RuminococcaceaeUCG-005/002,* and *(Eubacterium)xylanophylumgroup*. For the secondary BA UDCA, a positive association with *Dorea*, *Blautia*, *Agathobacter*, *Lachnoclostridium* and a negative association with *ChristensenellaceaeR-7 group* and *RuminococcaceaeUCG-005/003* was observed. These results support a distinct association profile of gut microbiota and bacterial metabolites between HC and ASD patients.

### 3.4. Transfer of FS–ASD in Mice Induces Changes in Colonic Permeability

Our results suggest that the fecal microbiota composition is modified in ASD patients, although no change in a limited number of metabolites was detected. Given that the integrity of microbiota is critical for GI functions, we sought to investigate whether microbiota-derived mediators present in FS could induce changes in GI functions in mice. To this aim, antibiotic-treated mice were subjected to enemas of FS from HC and ASD individuals, and their GI tract functions and integrity were studied.

**Figure 2:**
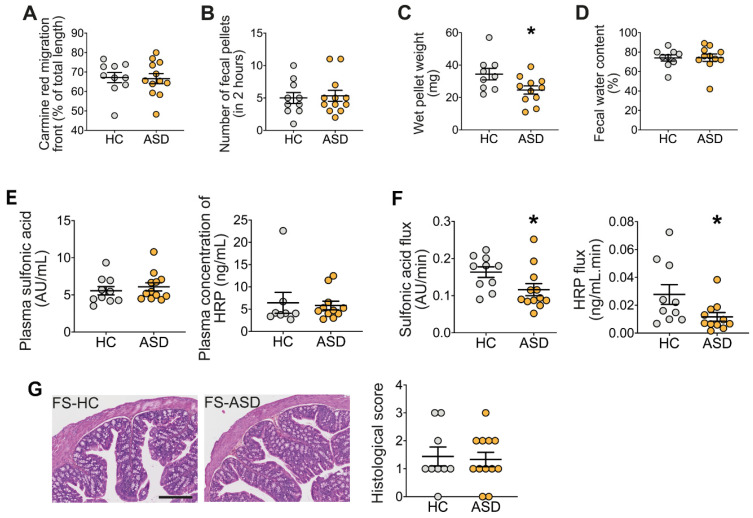
Transfer of FS–ASD to mice decreased pellet weight and reduced colonic permeability. (**A**) Intestinal transit rate measured as the distance of migration of carmine red over total intestinal length. (**B**) In vivo colonic propulsive motor function assessed by the measure of the number of pellets expelled for 2 h. (**C**) Total weight and (**D**) fecal water content of fecal pellets. All values represent means ± SEM (HC: *n* = 9–10; ASD: *n* = 11–12). Statistical analyses were performed with the Mann–Whitney U-test or Student *t*-test, * *p* < 0.05. (**E**) in vivo and (**F**) ex vivo paracellular (sulfonic acid) and transcellular permeability (horseradish peroxidase, HRP) in proximal colon segments. For (**F**), colonic permeability was determined in Ussing chambers by measuring the mucosal to serosal flux of the markers. All values represent means ± SEM (HC: *n* = 8–10; ASD: *n* = 11–12). Statistical analyses were performed with the Mann–Whitney U-test or Student *t*-test, * *p* < 0.05. (**G**) Morphological parameters of the proximal colon characterized by a histological score integrating quantification of the muscle thickening, mucosa integrity, and cellular infiltration (scale bar 250 µm).

First, the intestinal transit rate (assessed following carmine red gavage) was similar in mice treated with either FS–HC or FS–ASD (Figure 2A). The colonic transit, assessed by the fecal pellet output (FPO) measurements, was also similar between mice treated with FS–ASD or FS–HC (Figure 2B). However, the pellet wet weight was significantly reduced in mice treated with FS–ASD as compared to mice treated with FS–HC (Figure 2C), while the fecal water content was similar between the two groups (Figure 2D). We further evaluated the impact of FS enemas on in vivo paracellular, and transcellular permeability assessed with FSA and HRP as tracers, respectively. No difference in in vivo intestinal permeability to FSA or HRP was detected between mice treated with FS–HC and FS–ASD enemas (Figure 2E). However, in mice treated with FS–ASD, the ex vivo permeability to both FSA and HRP was reduced in the proximal colon but not distal colon, as compared to mice treated with FS–HC (Figure 2F, Additional file 5 Figure S3). We found that FS–ASD induced a 29% and 58% decrease for paracellular and transcellular permeability, respectively, compared to FS–HC. We next assessed colonic tissue integrity of the proximal colon. The histological score was similar in the colon following enemas of FS–HC or FS–ASD, revealing no difference in muscle thickness, mucosa integrity, or cellular infiltration (Figure 2G).

### 3.5. Transfer of FS–ASD in Mice Modulates Epithelial Barrier Integrity and Inflammation-Related Gene Expression in the Colon

**Figure 3:**
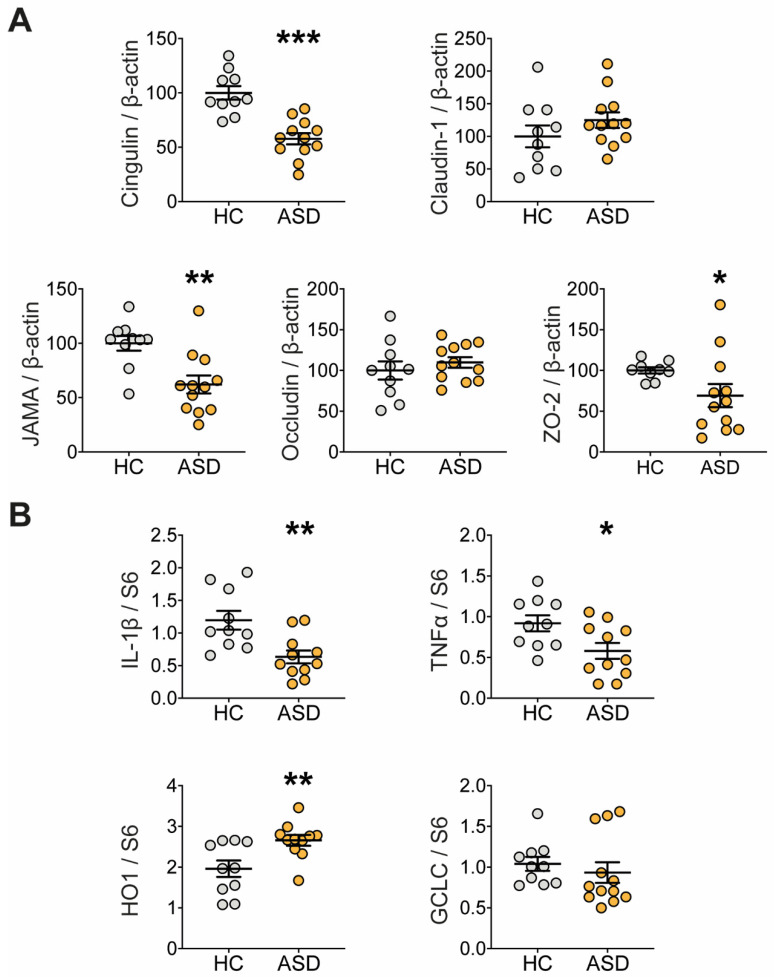
Transfer of FS–ASD modulates the expression of tight junction and inflammation-related molecules in the colon. (**A**) Protein expression of tight junction-forming molecules measured by Western blot in the proximal colon of mice treated with FS–HC or FS–ASD. For each protein, quantification of the signal intensity was normalized to theβ-actin signal of the same sample and expressed as a percentage of controls. (**B**) Gene expression of molecules with proinflammatory (IL-1β and TNFα) and anti-oxidative (HO-1 and GCLC) activity in the proximal colon of mice treated with FS–HC or FS–ASD. Western blot data are expressed as relative values to β-actin normalized to control, and q-PCR data are expressed as relative values to S6. All values represent means ± SEM (HC: *n* = 9–10; ASD: *n* = 11–12). Statistical analyses were performed with the Mann–Whitney U-test or Student *t*-test, * *p* < 0.05, ** *p* < 0.01, *** *p* < 0.001.

Changes in intestinal permeability are often associated with the altered expression of tight junction proteins and/or inflammatory markers [58]. We showed that the protein expression of key tight junction molecules was affected in the mice colon following treatment with FS–ASD in comparison to FS–HC (Figure 3A, Additional File 6 Table S3). In particular, the protein level of cingulin, Junctional Adhesion Molecule A (JAM-A), and ZO-2 were decreased in the proximal colon of FS–ASD compared to FS–HC mice while claudin-1 and occludin protein expression were unaffected (Figure 3A).

Next, we examined whether FS transfer modulated the gene expression of molecules involved in inflammatory and antioxidant responses (Figure 3B, Additional File 7 Table S4). As shown in Figure 3B, the gene expression of the pro-inflammatory cytokines IL-1β and TNFα was reduced in the proximal colon of FS–ASD-treated mice by 47 and 37%, respectively, when compared to the controls. Furthermore, mRNA expression of the antioxidant molecule heme oxygenase-1 (HO-1) was increased by 36% in mice treated with FS–ASD in comparison to FS–HC. Regarding glutamate-cysteine ligase catalytic subunit (GCLC), an enzyme involved in glutathione synthesis, no difference was found between the two groups (Figure 3B).

### 3.6. Transfer of FS–ASD in Mice Modulates Expression of Glial and Neuronal Molecules

**Figure 4:**
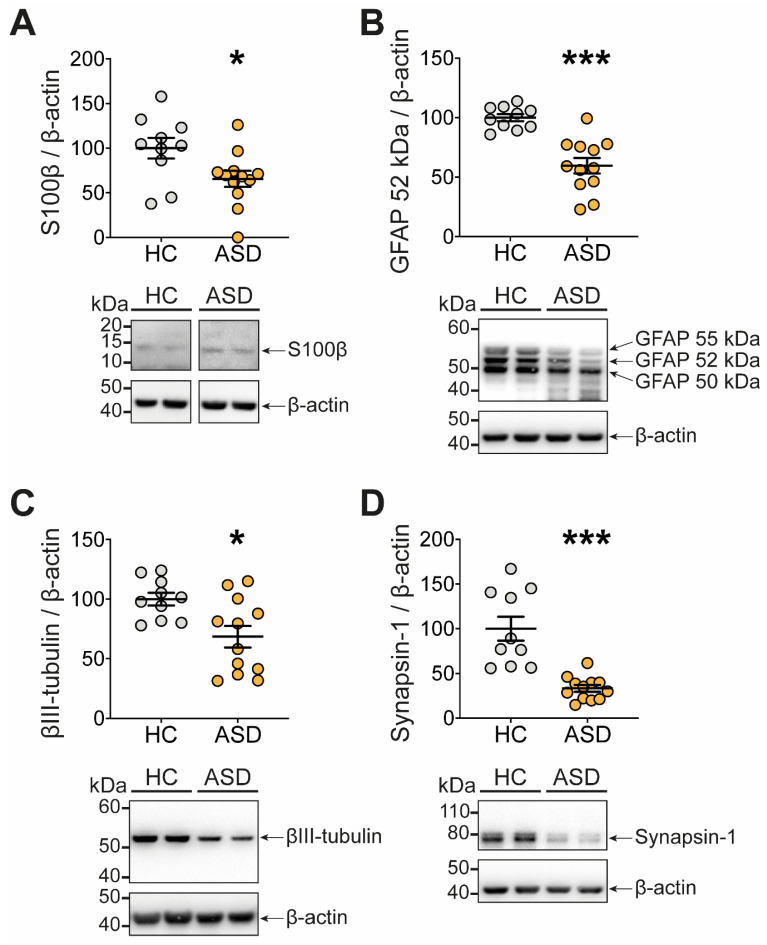
Transfer of FS–ASD to mice modulates the expression of glial and neuronal molecules. Protein expression of the glial molecules S100β (**A**) and GFAP (**B**) in the proximal colon of mice treated with FS–HC or FS–ASD. Protein expression of the neuronal molecules βIII-tubulin (**C**) and Synapsin 1 (**D**) in the proximal colon of mice treated with FS–HC or FS–ASD. Western blot data are expressed as relative values to β-actin normalized to control. All values represent means ± SEM (HC: *n* = 10; ASD: *n* = 12). Statistical analyses were performed with the Mann–Whitney U-test or Student *t*-test, * *p* < 0.05, *** *p* < 0.001.

As the ENS is a central regulator of gut functions, we investigated whether functional changes induced by FS–ASD in the proximal colon were associated with a remodeling of the ENS, which we assessed by analyzing the expression of key glial and neuronal molecules (Figure 4, Additional File 8 Table S5).

First, compared to FS–HC, FS–ASD induced a significant decrease in the protein expression of the glial molecules S100β (Figure 4A) and GFAP, though for the latter, it concerns only the 52 kDa form, while the 50 and 55 kDa forms were unaffected (Figure 4B).

Next, we aimed to determine the impact of FS–ASD upon key molecules involved in neuronal connectivity, i.e., βIII-tubulin, a major component of the neuronal microtubule network, and synapsin 1, a presynaptic protein associated with synaptic vesicles in axon terminals. Compared to FS–HC, FS–ASD decreased the protein expression of βIII-tubulin and synapsin 1 by 31% and 67%, respectively (Figure 4C,D). Altogether, our results indicated that the expression of critical molecules for ENS connectivity was specifically regulated by FS–ASD as compared to FS–HC.

### 3.7. FS Can Exert a Direct Effect on the ENS by Modulating Expression of Neuronal Molecules

**Figure 5:**
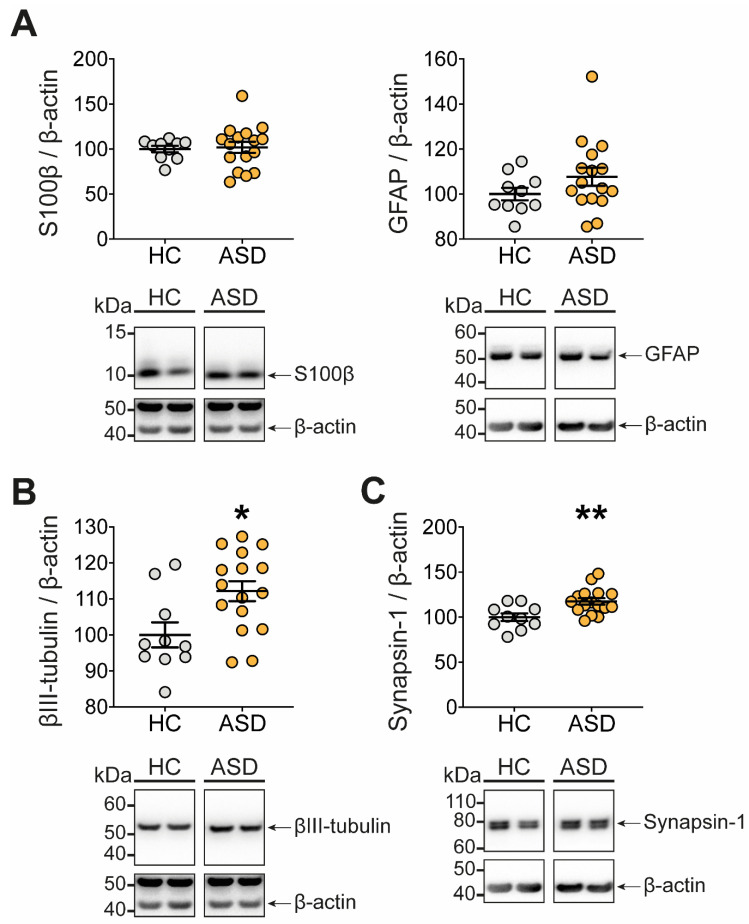
FS–ASD applied to ENS primary cultures induces a remodeling of glial and neuronal molecule expression. Protein expression of the glial molecules S100β and GFAP (**A**) in ENS cultures treated with FS–HC or FS–ASD. Protein expression of the neuronal molecules βIII-tubulin (**B**) and Synapsin 1 (**C**) in ENS cultures treated with FS–HC or FS–ASD. Western blot data are expressed as relative values to β-actin normalized to control. All values represent means ± SEM (HC: *n* = 10; ASD: *n* = 16). Statistical analyses were performed with the Mann–Whitney U-test or Student *t*-test, * *p* < 0.05, ** *p* < 0.01.

In the final step, we aimed to determine whether FS–ASD could directly remodel the ENS. Incubation of ENS primary cultures incubated with FS–HC or FS–ASD showed that the protein expression of S100β and GFAP were not impacted by FS–ASD treatment (Figure 5A, Additional File 9 Table S6). In contrast, the expression of βIII-tubulin and synapsin 1 were both increased by FS–ASD as compared to FS–HC (Figure 5B,C).

## 4. Discussion

This translational study provides novel and important evidence that (1) adult ASD patients present alterations in the fecal microbiota composition as compared to healthy controls, and (2) administration of FS from ASD patients induced changes in colonic functions and ENS phenotype, as compared to FS from HC. Altogether, our findings support the possibility that alterations of gut microbiota may participate in gut disturbances in ASD by, in part, inducing ENS remodeling.

While previous studies have highlighted changes in gut microbiota in children with ASD [34,35,36], our work demonstrated that microbiota alterations are also present in adults with ASD. We identified a reduced α-diversity in ASD patients compared to healthy subjects while β-diversity did not differ significantly. Our results support previous studies showing an association between ASD in children and less diverse microbiota overall [40,59], but contrast with other reports [17,31,57,60]. In our study, individuals with ASD exhibited a decreased ratio of *Firmicutes* to *Bacteroidetes* abundances in agreement with previous studies [31,57] but not others [40,60,61,62]. At the genus level, we identified three ASVs belonging to *Bacteroides*, *Desulfovibrio*, and *Ruminiclostridium* sp. that were increased in individuals with ASD, as previously described [31,33,57,62,63]. *Bacteroides*, *Desulfovibrio*, and some *Clostridia* genera have been associated with more severe autistic symptoms in children [31,63,64,65,66]. The few alterations of bacterial composition may reflect, in part, the fact that our study deals with a group of adults with ASD, as opposed to a pediatric population. While larger differences might occur in childhood, those could lessen with age, possibly due to the microbiota stabilization during aging and the influence of additional environmental factors such as nutrition and medication. Moreover, these results would need to be further investigated in a larger cohort of adults and using additional multiple differential abundance methods [52]. Furthermore, we analyzed a specific population of ASD patients, diagnosed with Asperger syndrome or autism without mental retardation. One could therefore speculate that our results reflect a microbiota profile assigned of this specific subgroup of ASD patients. The subtle alterations of bacterial composition observed in our study could also explain the lack of major changes in SCFA and BA concentrations measured in the FS of ASD as compared to HC. To date, no studies have examined the level of BA in ASD patients, but several studies have investigated the fecal concentration of SCFA in children with ASD. These later reported conflicting results such as no difference [67] or an increase in ASD children in comparison to controls [33]. Further studies would be required to identify other classes of bacterial metabolites possibly modified in adult ASD patients.

A second major finding of this study is the modification of the colonic epithelial barrier function induced by FS–ASD relative to FS–HC. We found that mice subjected to FS–ASD enemas displayed no change in intestinal permeability in vivo but a reduced ex vivo paracellular and transcellular permeability in the proximal but not in the distal colon. One should be cautious in interpreting the absence of effects of FS upon in vivo permeability as (i) in vivo permeability was assessed 2 h after gavage with FSA and reflects mainly small intestinal permeability, and (ii) the rectal enema used to administer FS preferentially irrigates the colon but not the small intestine [48]. The intestinal permeability assessed in vivo in various mouse models of ASD showed either an increase [39] or no modification [18,20]. In clinical studies, no difference [13] or increased intestinal permeability have been shown in ASD children in comparison to controls [11,12], while the colonic permeability has not been specifically addressed so far. In our study, the reduced colonic permeability was concomitant to an increased expression of the antioxidant molecule HO-1 and a reduced expression of inflammatory cytokines such as TNF-α and IL-1β. It is tempting to speculate that the increase in antioxidant response induced by FS–ASD could contribute to the reduced permeability by modulating the intestinal barrier microenvironment. Indeed, FS–ASD induced expression of HO-1 could be responsible (1) for the reduced expression of pro-inflammatory cytokines such as TNFα and IL-1β [68] which are known to increase paracellular and transcellular permeability [69,70], and (2) for regulating directly intestinal barrier function by modulating the expression of tight junction proteins [71]. Among mechanisms that could be involved in FS–ASD effects upon intestinal barriers are the decreased protein expression of the tight junction proteins ZO-2 and JAM-A. Indeed, decreased expression of ZO-2 and JAM-A was previously shown to enhance transepithelial electrical resistance [72] and intestinal epithelial cell proliferation [73,74,75], respectively, that might concomitantly contribute to the reduced colonic permeability observed in our study.

Another major finding of this translational study is the ability of FS–ASD to induce a remodeling of enteric neuronal and glial protein expression in mice colons as compared to FS–HC [69,70,71,72,73,74,75]. In particular, the decreased protein level of βIII-tubulin and synapsin 1 in mice treated with FS–ASD suggest that factors present in the FS from ASD patients might induce defects in the ENS neuronal function and synaptic connectivity. Although anomalies in the ENS connectivity have been shown to result in bowel dysmotility [22,76], no changes in total transit time and colonic transit were reported in our study. One possibility is that FS–ASD might affect ENS and therefore motility functions in an organ-specific manner. While we found significant changes in the expression of ENS molecules in the proximal colon, the other parts of the gut that were not assessed in our study, namely the small intestine and distal colon, might be unaffected by FS–ASD, resulting thereby, in normal basal motility function. Another possibility is that ENS defects induced by FS–ASD would not affect basal motility function, but only challenged condition, such as the presence of a stressor, receptor stimulation or blockade, would reveal the consequences of ENS disturbance upon gut functions. For instance, in a Neuroligin-3 genetic mouse model of ASD, no change in the frequency of motor activity was observed in the colon under basal conditions, but exposure to GABA receptor antagonists reduced the colonic motility [19]. Interestingly, the decreased expression of neuronal connectivity proteins was concomitant to a reduced level of the enteric glial proteins S100β and GFAP in mice treated with FS–ASD as compared to FS–HC. Considering that enteric glial cells have been shown to promote neuronal network complexity and synapse number [77], it is tempting to speculate that modifications of enteric glial cells could, at least in part, contribute to the altered expression of the neuronal connectivity-related molecules. A final part of our study was to examine in ENS primary cultures whether FS–ASD could directly impact the ENS. We found that, in vivo, βIII-tubulin and synapsin 1 were regulated by SF–ASD, but oppositely. The cause for this differential regulation remains unknown but suggests that the cellular environment of the ENS could be critical to mediate FS-induced ENS remodeling. In particular, gut-resident cell types absent in our ENS primary culture model, such as intestinal epithelial and immune cells, could contribute to ENS remodeling in response to FS–ASD. The use of more complex models, such as the coculture of ENS cells with intestinal epithelial or/and immune cells or intestinal organoids with ENS [78] could help unravel the contribution of specific cell types upon the ENS response observed in vivo.

The modifications induced by FS–ASD on the expression of molecules related to ENS neuronal connectivity are of particular interest given that anomalies in brain neuronal connectivity have been reported to be associated with ASD [5,6,7,79]. One could hypothesize that factors present in FS–ASD contribute to mirror in the ENS the changes in neuronal connectivity reported in the brain. Supporting this hypothesis are the concomitant brain and ENS alterations reported in genetic animal models of ASD [23].

In conclusion, our translational study demonstrated the ability of FS from ASD individuals, as compared to HC, to induce GI and ENS alterations in mice. Our results support the model that microbiota could contribute to pathophysiological mechanisms underlying ASD-associated GI symptoms. Future studies focusing on the identification of microbiota-derived metabolites responsible for these effects might result in innovative intervention in ASD.

## Figures and Tables

**Table 1:** Fecal metabolite concentrations of HC and ASD individuals.

	HC	(*n*)	ASD	(*n*)	*p* Values
SCFA (mM)					
Acetate	6.57 ± 0.61	(15)	7.36 ± 0.47	(38)	*p* = 0.356
Propionate	2.35 ± 0.28	(15)	2.57 ± 0.19	(37)	*p* = 0.494
Butyrate	1.98 ± 0.19	(15)	2.61 ± 0.25	(38)	*p* = 0.276
Isobutyrate	0.29 ± 0.04	(15)	0.31 ± 0.03	(38)	*p* = 0.715
Valerate	0.26 ± 0.02	(14)	0.30 ± 0.02	(38)	*p* = 0.688
Bile acids (µg/mL)					
Primary					
CDCA	0.045 ± 0.011	(13)	0.050 ± 0.007	(37)	*p* = 0.747
CA	0.006 ± 0.002	(13)	0.009 ± 0.002	(32)	*p* = 0.492
Secondary					
DCA	14.83 ± 4.24	(13)	26.87 ± 3.57	(37)	*p* = 0.057
LCA	0.710 ± 0.162	(12)	0.537 ± 0.075	(30)	*p* = 0.466
UDCA	0.030 ± 0.007	(12)	0.043 ± 0.009	(25)	*p* = 0.471

Concentration of short chain fatty acids and bile acids in the fecal supernatant of control subjects (HC) and ASD individuals. The values in parenthesis denote the number of individuals in each group. Patients for which metabolites were not detected by our method were removed. All values represent means ± SEM. Statistical analyses were performed with the Mann–Whitney U-test or Student *t*-test.

## Data Availability

The data presented in this study are available on request from the corresponding authors.

## Supplementary Materials

The following are available online at https://www.mdpi.com/article/10.3390/microorganisms9081723/s1, Additional File 1: Table S1. Listing of primers used in this study. Additional File 2: Table S2. Listing of antibodies used in this study. Additional File 3: Figure S1. Microbial β-diversity and relative abundance in HC and ASD individuals. (A–C) Principal coordinate analysis (PCoA) plots of fecal samples of HC and ASD individuals. Plots were generated according to: (A) Jaccard, (B) Unweighted UniFrac, and (C) Weighted UniFrac distance matrix. (D,E) Clustering of samples and their organization according to experimental factors. (D) NMDS representation of samples colored by cluster and shaped with the most influential factor (sex). (E) influence of each factor in each cluster by using the Normalized Mutual Information (NMI). (F,G) Bacterial taxonomic profile in all HC and ASD individuals at phylum (F) and genus (G) level. (H) Top 5 most abundant genus present in HC and ASD individuals. Statistical analyses were performed with the Wilcoxson rank test. Additional File 4: Figure S2. Correlation between fecal microbiota and metabolites in HC and ASD individuals. Heatmap of Spearman correlation of fecal bacterial genera and metabolites in HC (A) and ASD (B) individuals. Bacterial genera are represented on the *x-axis*, and microbiota-derived short chain fatty acids and bile acids are represented on the *y-axis*. Red squares correspond to positive Spearman correlations while negative correlations are shown in blue. Statistical analyses were generated using the associate function of microbiome package. Only results displaying an adjusted *p*-value below 0.05 after Benjamini–Hochberg correction (FDR) are reported. Additional File 5: Figure S3. Transfer of FS–ASD to mice had no effect on distal colonic permeability. Ex vivo paracellular (sulfonic acid) and transcellular (horseradish peroxidase, HRP) permeability in distal colon segments. Colonic permeability was determined in Ussing chambers by measuring the mucosal to serosal flux of the markers. All values represent means ± SEM (HC: *n* = 8–10; ASD: *n* = 11–12). Statistical analyses were performed with the Mann–Whitney U-test. Additional File 6: Table S3. Transfer of FS–ASD to mice modulates the expression of tight junction components in the colon. (A) Quantification of the mRNA expression of epithelial barrier genes in the proximal colon of mice treated with FS–HC or FS–ASD. (B) Quantification of the expression of epithelial barrier proteins in the proximal colon of mice treated with FS–HC or FS–ASD. Western blot data are expressed as relative values to β-actin normalized to control, and q-PCR data are expressed as relative values to S6. All values represent means ± SEM (HC: *n* = 9–10; ASD: *n* = 10–12). Statistical analyses were performed with the Mann–Whitney U-test or Student t-test. Additional File 7: Table S4. Transfer of FS–ASD to mice modulates the expression of inflammation-related molecules in the colon. (A) Quantification of the mRNA expression of inflammatory genes in the proximal colon of mice treated with FS–HC or FS–ASD. (B) Quantification of the mRNA expression of genes involved in oxidative stress in the proximal colon of mice treated with FS–HC or FS–ASD. q-PCR data are expressed as relative values to S6. All values represent means ± SEM (HC: *n* = 10; ASD: *n* = 11–12). Statistical analyses were performed with the Mann–Whitney U-test or Student *t*-test. Additional File 8: Table S5. Transfer of FS–ASD to mice modulates the expression of glial and neuronal molecules. (A) Quantification of the mRNA expression of glial and neuronal genes in the proximal colon of mice treated with FS–HC or FS–ASD. (B) Quantification of the expression of glial and neuronal proteins in the proximal colon of mice treated with FS–HC or FS–ASD. Western blot data are expressed as relative values to β-actin normalized to control, and q-PCR data are expressed as relative values to S6. All values represent means ± SEM (HC: *n* = 10; ASD: *n* = 12). Statistical analyses were performed with the Mann–Whitney U-test or Student *t*-test. Additional File 9: Table S6. FS–ASD applied to ENS primary cultures induces a remodeling of glial and neuronal molecule expression. (A) Quantification of the mRNA expression of glial and neuronal genes in ENS cultures treated with FS–HC or FS–ASD (*n* = 6 cultures). (B) Quantification of the mRNA expression of glial and neuronal genes in ENS cultures treated with FS–HC or FS–ASD (*n* = 4 cultures). (C) Quantification of the expression of glial and neuronal proteins in ENS cultures treated with FS–HC or FS–ASD (*n* = 6 cultures). Western blot data are expressed as relative values to β-actin normalized to control, and q-PCR data are expressed as relative values to S6. All values represent means ± SEM (HC: *n* = 10; ASD: *n* = 15–16). Statistical analyses were performed with the Mann–Whitney U-test or Student *t*-test.
