# Supplementary figures and images for: Fecal Supernatant from Adult with Autism Spectrum Disorder Alters Digestive Functions, Intestinal Epithelial Barrier, and Enteric Nervous System
Source: Microorganisms. 2021 Aug 13;9(8):1723. doi: 10.3390/microorganisms9081723 (PMC8399841; doi:10.3390/microorganisms9081723)

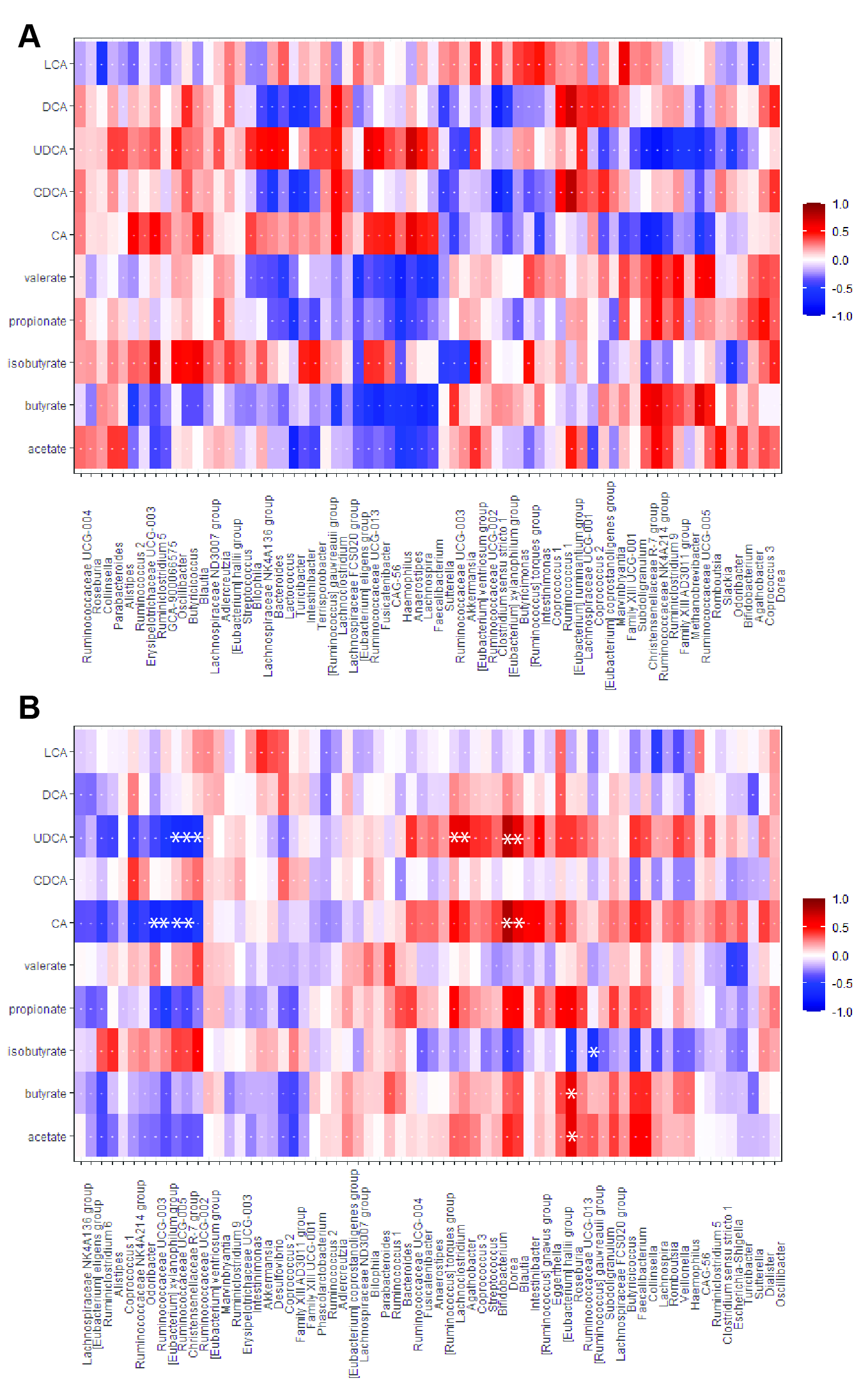

Supplement: Supplementary file 1 [file microorganisms-09-01723-s001.zip › Additional file 4 Figure S2.tif]

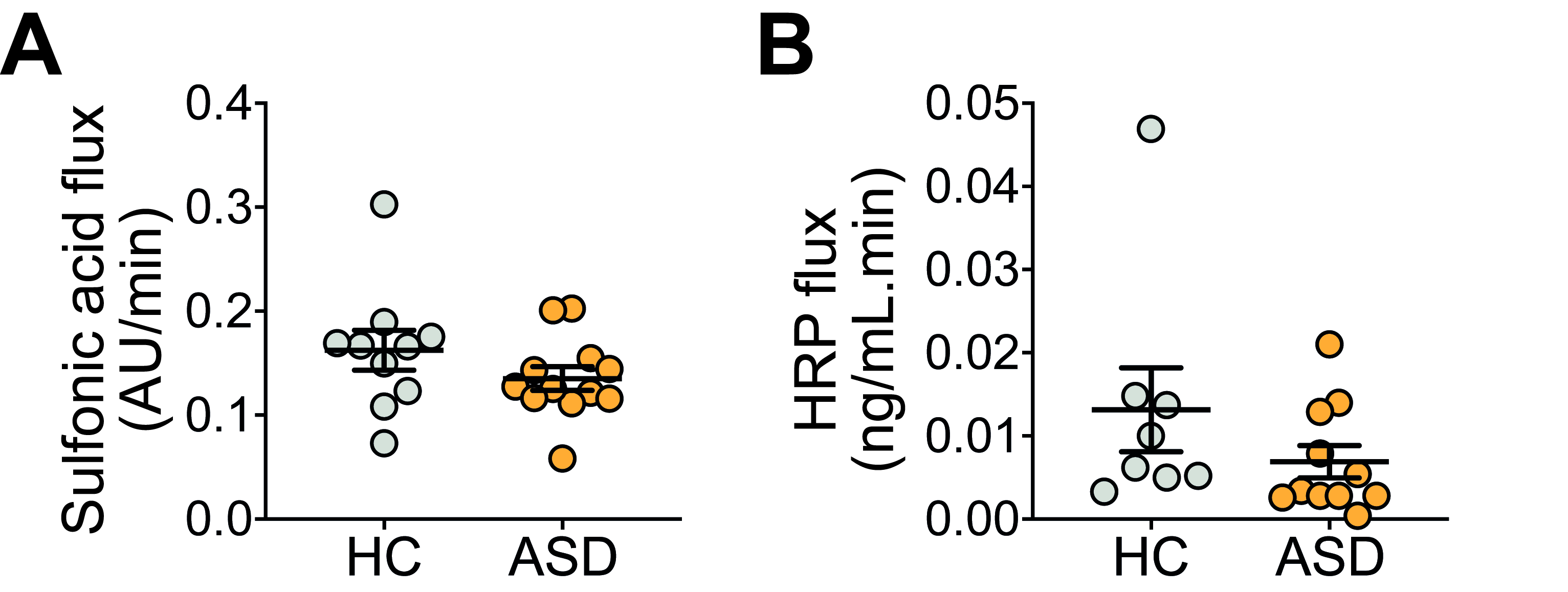

Supplement: Supplementary file 1 [file microorganisms-09-01723-s001.zip › Additional file 5 Figure S3.tif]

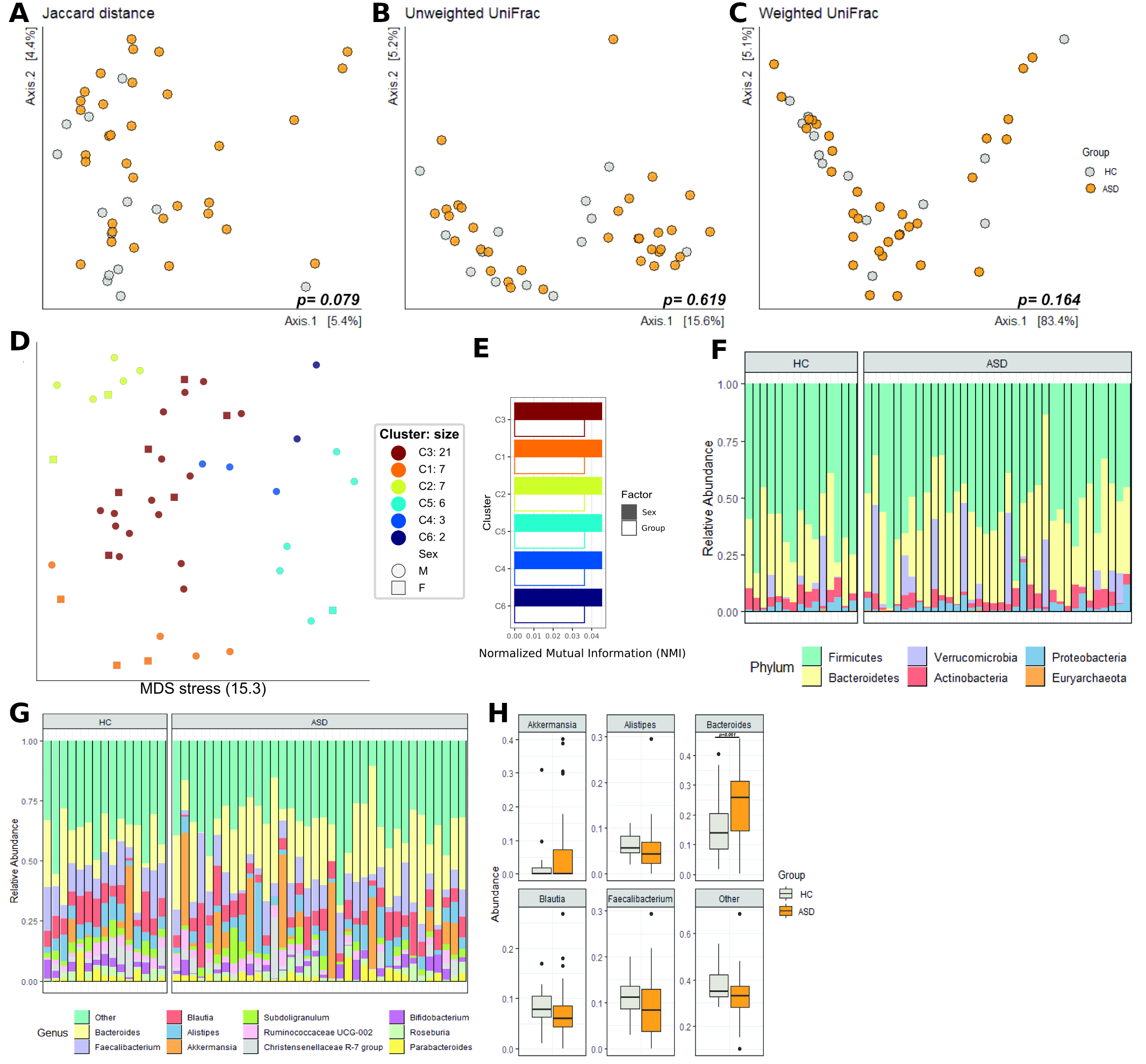

Supplement: Supplementary file 1 [file microorganisms-09-01723-s001.zip › Additional file 3 Figure S1.tif]
